# Computerized cognitive training and brain derived neurotrophic factor during bed rest: mechanisms to protect individual during acute stress

**DOI:** 10.18632/aging.101166

**Published:** 2017-02-03

**Authors:** Angelina Passaro, Cecilia Soavi, Uros Marusic, Enrico Rejc, Juana M. Sanz, Mario L. Morieri, Edoardo Dalla Nora, Voyko Kavcic, Marco V. Narici, Carlo Reggiani, Gianni Biolo, Giovanni Zuliani, Stefano Lazzer, Rado Pišot

**Affiliations:** ^1^ Medical Science Department, University of Ferrara, Ferrara 44121, Italy; ^2^ Science and Research Centre, University of Primorska, Koper 6000, Slovenia; ^3^ Kentucky Spinal Cord Injury Research Center, University of Louisville, Louisville, KY 40202, USA; ^4^ Institute of Gerontology, Wayne State University, Detroit, MI 48202, USA; ^5^ Biomedical Research and Innovative Society, Ljubljana 1000, Slovenia; ^6^ School of Graduate Entry Medicine and Health, University of Nottingham, Derby DE22 3NE, UK; ^7^ Department of Biomedical Sciences, University of Padua, Padua 35131 Italy; ^8^ Department of Medical, Surgical and Health Sciences, Division of Internal Medicine, University of Trieste, Trieste 34149, Italy; ^9^ Department of Medical and Biological Sciences, University of Udine, Udine 33100, Italy; ^10^ School of Sport Sciences, University of Udine, Udine 33100, Italy

**Keywords:** acute stress, aging, BDNF, bed rest, cognitive training, metabolism

## Abstract

Acute stress, as bed rest, was shown to increase plasma level of the neurotrophin brain-derived neurotrophic factor (BDNF) in older, but not in young adults. This increase might represent a protective mechanism towards acute insults in aging subjects. Since computerized cognitive training (CCT) is known to protect brain, herein we evaluated the effect of CCT during bed rest on BDNF, muscle mass, neuromuscular function and metabolic parameters. The subjects that underwent CCT did not show an increase of BDNF after bed rest, and showed an anti-insular modification pattern in metabolism. Neuromuscular function parameters, already shown to beneficiate from CCT, negatively correlated with BDNF in research participants undergoing CCT, while positively correlated in the control group. In conclusion, BDNF increase can be interpreted as a standardized protective mechanism taking place whenever an insult occurs; it gives low, but consistent preservation of neuromuscular function. CCT, acting as an external protective mechanism, seems to modify this standardized response, avoiding BDNF increase or possibly modifying its time course. Our results suggest the possibility of differential neuroprotective mechanisms among ill and healthy individuals, and the importance of timing in determining the effects of protective mechanisms.

## INTRODUCTION

Elderly individuals are often bedridden at a certain point of their life (e.g. acute medical condition needing hospitalization) with negative effects on metabolism, neuromuscular and cognitive performance. Bed rest induces insulin resistance, increases cholesterol and triglyceride plasma levels, leads to hypertension [1, 2]; it also induces neuromuscular dysfunction within few weeks, substantially affecting muscle strength and power exertion [3, 4]. Moreover, bed rest acts as a stressful agent on brain, negatively affecting brain structure [5], electro-cortical activity [6], executive functions, and mood [7, 8].

Brain-derived neurotrophic factor (BDNF), growth factor member of the neurotrophin family, binds specifically to the Tropomiosine receptor kinase B (Trk B) mediating neurotrophic signaling [9]. During developement it plays a critical role in cell differentiation, migration, neuronal survival, dendritic arborisation, synaptogenesis, and synaptic plasticity. In adult brain BDNF plays a role in damage repairing and resistance to insults [9]. Crossing bi-directionally the blood-brain barrier, BDNF can act at a systemic level. Low levels of plasma BDNF have been associated with dementia [10, 11], diabetes [11–13], depression [14, 15] and coronary syndrome [16].

At muscular level, muscle BDNF can increase fatty acid oxidation, ameliorating energy afflux in an autocrine/paracrine manner [17]. Many studies demonstrate that physical activity increases circulating BDNF in humans [18-25], however this increase is attributed to an increase in cerebral production, which accounts for 70-80% of total circulating BDNF both at rest and after physical activity [21]. We have already studied plasma BDNF modification in response to immobilization, both in young and older adults [26], demonstrating an increase of BDNF level only in the latter group. We hypothesized that this increase might represent the effort of older brain, less resistant to acute stress, to counteract bed rest negative effects. The concept that bed rest represents a considerable stress is confirmed by the increases in cortisol level [27, 28].

Previous studies showed that computerized cognitive training (CCT) can promote higher preservation of cognitive function [29] and can improve mobility control in daily life [30] as well as during bed rest [31] in healthy elderly people. CCT also improved mood in depressive patients [32], memory and attention in stroke patients [33], and gait and possibly cognition in Parkinson's and Alzheimer's, and brain injured patients [34].

The aim of the present study was to evaluate the combined effect of CCT and 14 days of horizontal bed rest on plasma BDNF levels, anthropometric metabolic parameters and neuromuscular function, in older adult volunteers.

## RESULTS

Table 1 shows the characteristics of the two groups (CCT vs noCCT) at baseline data collection (BDC). No differences were found in demographic/anthropometric characteristics, metabolic and inflammatory/stress response profile, nor in muscular or integrated function parameters.

**Table 1:** Baseline characteristics of the study population. Data are expressed as mean ± standard deviation

	noCCT (N 8)	CCT (N 8)	P
Demographic and Anthropometric characteristics			
Age (years)	59.1±2.5	59.1±3.6	0.991
BMI (Kg/mq)	26.9±4.2	26.9±5.2	0.928
FFM (Kg)	59.8±6.9	64.5±11.7	0.355
FM (Kg)	19.9±4.9	16.8±7.4	0.360
Metabolic profile			
Total cholesterol (mg/dl)	212.4±24.4	193.8±53.5	0.393
LDL cholesterol (mg/dl)	143.0±18.6	134.6±47.2	0.648
HDL cholesterol (mg/dl)	42.1±7.7	37.8±5.2	0.648
Triglycerides (mg/dl)	136.5±33.9	107.6±51.9	0.219
Insulin (mU/I)	5.7±1.1	6.3±4.4	0.720
Glucose (mg/dl)	79.6±9.5	76.5±7.5	0.489
HOMA IR	1.12±0.27	1.15±0.88	0.812
Inflammation/stress markers			
C-reactive protein (mg/dl)	0.11±0.06	0.12±0.13	0.815
TNF-alpha	1.45±2.65	4.35±5.60	0.211
BDNF (pg/ml)	37.53±18.58	47.81±18.57	0.305
Muscular parameters			
QF muscle volume (cm^3^)	1663.8±173.4	1755.9±277.1	0.447
BCM (Kg)	30.90±4.75	33.84±7.01	0.353
MM (Kg)	38.28±5.48	41.81±8.41	0.346
MEP (W)	2653.5±566.8	2576.7±519.2	0.790
MVC (N)	564.4±120.9	524.1±112.2	0.518

BCM: body cellular mass; BDNF: brain derived neurotrophic factor; BMI: body mass index; CCT: computerized cognitive training; FFM: fat-free mass; FM: fat mass; HDL: high density lipoprotein; HOMA: homeostatic model assessment; LDL: low density lipoprotein; MEP: maximal explosive power of the lower limbs; MM: muscle mass; MVC: maximal voluntary contraction of knee extensors; QF: quadriceps femoris; TNF: tumour necrosis factor.

### Effect of bed rest on BDNF

**Figure 1:**
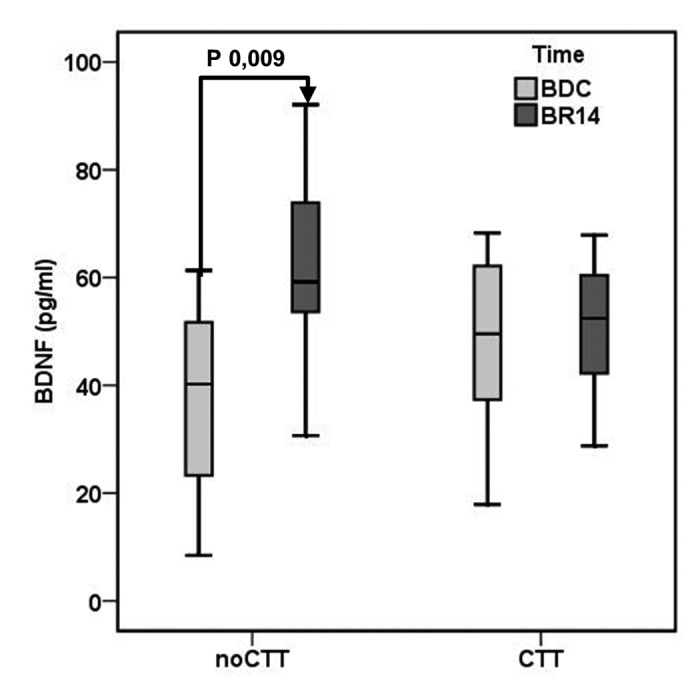
Mean brain-derived neurotrophic factor (BDNF) plasma levels in Computerized Cognitive Training (CCT) group and a no-Computerized Cognitive Training (noCCT) group at BDC (baseline data collection) and BR14 (14th day of bed rest) There was a significant interaction effect between time and group.

After 14 day of bed rest (BR14) a significant increase in plasma BDNF was observed in the noCCT group (BDC 37.53±18.58, BR14 62.02±18.31 pg/ml, P 0.009) but not in the CCT group (BDC 47.81±18.57, BR14 50.62±14.69 pg/ml, P 0.372) (Figure 1).

### Effect of bed rest on anthropometric-metabolic variables

The effect of both bed rest and CCT on anthropometric and metabolic variables are shown in Table 2. The “time effect” indicates the effect of undergoing bed rest, while “group effect” indicates the effect of undergoing CCT or not. A significant effect of time (i.e. undergoing bed rest) was observed for BMI, fat free mass, total and LDL cholesterol; moreover, no time x group interaction effect was demonstrated.

**Table 2:** Time and time x group interaction effect on anthropometric and metabolic variables

	Time effect			Time x Group effect		
	F	P	Partial η^2^	F	P	Partial η^2^
BMI	47.898	**<0.001**	0.787	1.264	0.281	0.089
FFM	17.009	**0.001**	0.567	0.470	0.505	0.035
FM	3.373	0.089	0.206	0.098	0.759	0.008
Total cholesterol	18.232	**0.001**	0.584	0.170	0.687	0.013
LDL cholesterol	15.597	**0.002**	0.545	0.631	0.441	0.046
HDL cholesterol	0.003	0.959	<0.001	4.134	0.063	0.241
Triglycerides	2.044	0.176	0.136	1.081	0.317	0.077
Insulin	4.236	0.062	0.261	0.275	0.610	0.022
Glucose	2.900	0.112	0.182	0.594	0.455	0.044
HOMA IR	0.115	0.741	0.009	0.790	0.392	0.062
C-reactive protein	2.105	0.171	0.139	0.640	0.438	0.047
TNF-alpha	2.581	0.132	0.166	2.127	0.168	0.141

Bold font is used to underline statistically significant results. BMI: body mass index; FFM: fat-free mass; FM: fat mass; HDL: high density lipoprotein; HOMA: homeostasis model assessment; LDL: low density lipoprotein; TNF: tumour necrosis factor.

Table 3 shows results of the paired sample T-test for anthropometric and metabolic variables. In both groups, a decrease in BMI at BR14 was observed, while only in the noCCT group a decrease in fat-free mass and an increase in fat mass were observed. LDL cholesterol was reduced at BR14 in both groups, while total cholesterol decreased significantly only in noCCT group. A reduction in triglycerides was noticed in noCCT group, while in CCT group an increase in glucose was observed.

**Table 3:** Paired sample T-test regarding anthropometric/metabolic variables. Data are expressed as mean ± standard deviation

	noCCT (N 8)		P	CCT (N 8)		P
	*BDC*	*BR14*		*BDC*	*BR14*	
Anthropometric characteristics						
BMI (Kg/mq)	26.9±4.2	26.2±4.1	**0.003**	26.6±5.2	25.7±4.7	**0.002**
FFM (Kg)	59.8±6.9	56.7±6.8	**0.000**	64.5±11.7	60.1±8.1	0.062
FM (Kg)	19.9±4.9	20.9±4.8	**0.002**	16.8±7.4	18.3±9.4	0.349
Metabolic profile						
Total cholesterol (mg/dl)	212.4±24.4	183.5±33.5	**0.006**	193.8±53.5	170.0±49.6	0.055
LDL cholesterol (mg/dl)	143.0±18.6	124.2±30.2	**0.031**	134.6±47.2	106.3±51.9	**0.030**
HDL cholesterol (mg/dl)	42.1±7.7	36.6±9.5	0.244	37.8±5.2	42.9±9.0	0.100
Triglycerides (mg/dl)	136.5±33.9	113.7±29.0	**0.034**	107.6±51.9	104.0±20.3	0.840
Insulin (mU/I)	5.69±1.11	4.85±1.57	0.106	6.60±4.66	6.06±4.30	0.313
Glucose (mg/dl)	79.6±9.5	82.4±15.0	0.622	76.5±7.5	83.8±6.1	**0.003**
HOMA IR	1.12±0.27	1.02±0.44	0.469	1.27±1.01	1.32±1.05	0.535
Inflammation/stress markers						
C-reactive protein (mg/dl)	0.11±0.06	0.36±.64	0.275	0.12±0.13	1.00±2.14	0.315
TNF-alpha	1.45±2.65	1.59±2.98	0.319	4.35±5.60	7.38±10.38	0.203

Bold font is used to underline statistically significant results. BDC: baseline data collection; BMI: body mass index; BR14: at 14^th^ day of bed rest; CCT: computerized cognitive training; FFM: fat-free mass; FM: fat mass; HDL: high density lipoprotein; HOMA: homeostasis model assessment; LDL: low density lipoprotein; TNF: tumour necrosis factor.

### Effect of bed rest on muscle mass and neuromuscular function

A significant time effect, but no time x group interaction effect, was observed for all variables concerning muscle quantity and function (Table 4). As shown in Table 5, in both groups bed rest induced a decrease in muscle mass parameters. Considering muscular function, a decrease in maximal explosive power of lower limbs was observed in both groups, while a reduction in maximal voluntary contraction was noticed only for noCCT group.

**Table 4:** Time and time x group interaction effect on muscle mass and neuromuscular function

	Time effect			Time x Group effect		
	F	P	Partial η^2^	F	P	Partial η^2^
QF muscle volume	79.388	**<0.001**	0.859	0.152	0.703	0.012
BCM	6.326	**0.026**	0.327	0.149	0.706	0.011
MM	7.601	**0.016**	0.369	0.192	0.669	0.015
MEP	23.167	**<0.001**	0.641	0.023	0.881	0.002
MVC	13.390	**0.003**	0.507	1.472	0.247	0.102

Bold font is used to underline statistically significant results. BCM: body cellular mass; MEP: maximal explosive power of lower limb; MM: muscle mass; MVC: maximal voluntary contraction of the knee extensors; QF: quadriceps femoris.

**Table 5:** Paired sample t-test regarding muscle mass and neuromuscular function variables. Data are expressed as mean ± standard deviation

	noCCT (N 8)		P value	CCT (N 8)		P value
	*BDC*	*BR14*		*BDC*	*BR14*	
Quantity measures						
BCM (Kg)	30.9±4.8	29.4±4.3	**<0,001**	33.8±7.0	31.8±3.7	**0.003**
MM(Kg)	38.3±5.5	36.4±5.1	**<0.001**	41.8±8.4	39.2±4.6	**0.003**
QF muscle volume (cm^3^)	1663.8±173.4	1524.3±196.9	**<0.001**	1755.9±277.1	1603.6±233.4	**<0.001**
Function measures						
MEP (W)	2653.5±566.8	2270.0±571.3	**0.021**	2576.7±519.2	2216.7±441.4	**0.001**
MVC (N)	564.4±120.9	519.6±123.9	**0.003**	524.1±112.2	435.0±80.9	0.104

Bold font is used to underline statistically significant results. BCM: body cellular mass; BDC: baseline data collection; BR14: at 14^th^ day of bed rest; CCT: computerized cognitive training; MEP: maximal explosive power of lower limb; MM: muscle mass; MVC: maximal voluntary contraction of the knee extensors; QF: quadriceps femoris.

### Correlation between BDNF and neuromuscular parameters

**Figure 2:**
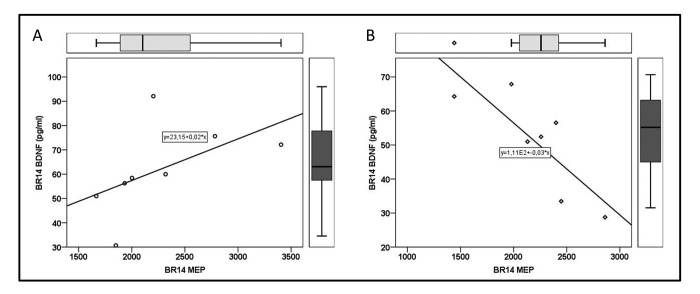
Correlation between brain-derived neurotrophic factor (BDNF) and maximal explosive power of lower limbs (MEP) on 14th day of bed rest (BR14) (**A**) no-Computerized Cognitive Training (noCCT) group, and (**B**) Computerized Cognitive Training (CCT) group.

No significant correlation between BDNF levels and neuromuscular parameters were found in the whole population at BDC. However, at BR14, BDNF positively correlated with maximal explosive power (MEP) in the noCCT group (R 0.810, P 0.015), and negatively in the CCT group (R -0.821, P 0.023) (Figure 2). Moreover, only in noCCT group, the variation of BDNF (ΔBDNF) negatively correlated with the variation of maximal voluntary contraction of the knee extensors (ΔMVC; R -0.905, P 0.002).

## DISCUSSION

When we designed this and the previous study [26], we hypothesized that we would have found a decrease in BDNF in people undergoing bed rest, and that this decrease would have been less important in people undergoing cognitive training, based on data coming from literature. The results were surprising, showing exactly the opposite. So, first, we had to understand the reason of the discrepancy between our result and results from literature.

We have previously demonstrated that bed rest induces an increase in plasma BDNF in adult/older, but not in young people [26]. Studies on animals [35] and on humans affected by diabetes and Alzheimer's disease [36, 37 ] seem to suggest a biphasic behaviour of BDNF with increased level in the first phase of disease and decreased level in a later phase. BDNF might increase to protect brain form an insult; however when the stressful event overcomes a certain threshold, resulting in a brain damage, cerebral production of BDNF might decrease, with a reduction of plasma BDNF. In line with this hypothesis, we speculated that, with aging, the brain might become less resistant to acute stresses; thus, the increase in plasma BDNF would represent the effort of the brain to counteract the stress induced by bed rest [26].

In the present study, we found that the increase in BDNF is not observed in adult-older subjects performing CCT during bed rest. We hypothesize that CCT might activate alternative protective pathways, overcoming the necessity of a BDNF increase. We can only speculate what mechanisms are involved. Possibly, CCT might induce a modulation of the expression of BDNF, its precursors and receptors. Animal studies have already shown that spatial memory training can affect the Trk B gene expression, and the proBDNF/BDNF ratio at hyppocampal level, with an increase of proBDNF but not of BDNF in older rats [38]. Since the preferential ligand of proBDNF is p75NTR [39], memory training might also modulate p75NTR pathway. In the animal, p75NTR is implicated in learning process, since knockout mice show very poor performances at Morris’ maze compared with controls [40]. Moreover p75NTR counteracts Trk B-mediated BDNF effect on glutamate release at a pre-synaptical level [41].

**Figure 3:**
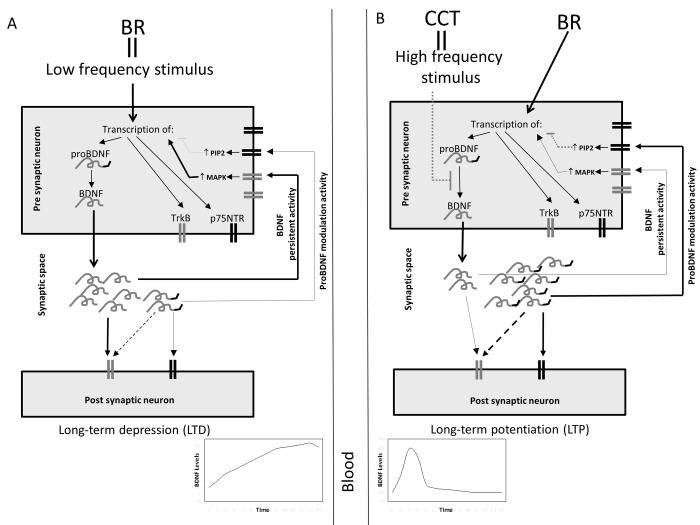
Schematic hypothetical representation of the possible effects of bed rest and CCT on BDNF release (**A**) Bed rest (BR) might act as a low-frequency stimulus inducing, at pre-synaptical level, the transcription of p75NTR, TrkB and proBDNF, which is proteolytically converted in BDNF. Thus, in the synaptical space might be released a high amount of mature BDNF and a low amount of proBDNF; both at post-synaptical and at pre-synaptical level, these molecules bind their specific receptors. BDNF binds to TrkB; at pre-synaptical level this induces the activation of MAPK pathway which, in turn, maintains active the transcription of BDNF. ProBDNF binds minimally to TrkB and principally to p75NTR; its activation at pre-synaptical level, via PIP2 pathway, leads to the suppression of BDNF transcription. Since BDNF amount is higher than proBDNF, the final balanced effect might be the persistant BDNF transcription and activity. This mechanism could be responsible for an increase of BDNF level in synaptic space and consequently in circulation. (**B**) CCT, acting as an high frequency stimulus, might be responsible for blocking the maturation of BDNF, leading to the release of high amounts of proBDNF and low amounts of BDNF in the synaptic space. In this case proBDNF modulatory effect might prevail on BDNF's leading, in turn, to the suppression of the transcription of BDNF. In this case a rapid and transient increase of BDNF in the synaptic space and in circulation might occur.

The reason of the differential expression of these molecules might be the frequency of stimulation of the presynaptic cell. It is known that BDNF plays a crucial role in long term potentiation process [42], and that glutamate receptor NMDA is strictly involved in this process [43]. NMDA receptors are implicated both in long term potentiation and depression, based on the frequency of presynaptic stimulation [44]. Possibly, in people undergoing CCT during bed rest, the repeated cognitive stimulation might early induce the fixation of the acquired skills via long term potentiation. A preferential increase of proBDNF might activate p75NTR receptors, inducing their modulatory activity and secondary leading to a reduction of BDNF release. Rapanelli et al. [45] seems to sustain this hypothesis: in their work, in the early phase of training, “learning mice” have hippocampal BDNF levels much higher compared with “trained mice”, that at the end of training program have learned skills. This seems to indicate that, when a task is learned, cellular activation persists but plasticity declines. The above mentioned hypothetical mechanism underlying the observed results is represented in the schematic diagrams reported in Figure 3.

### Bed rest, computerized cognitive training, and anthropometric-metabolic parameters

We evaluated how bed rest influenced anthropometric, metabolic, and inflammatory variables in CCT and noCCT groups. Behind the expected reduction in BMI in both groups, secondary to muscular atrophy induced by bed rest [46-48], we found important differences.

People performing CCT showed an increase in glucose levels; on the opposite, some modifications seen in noCCT group, (the increase in fat mass and the decrease in triglycerides) did not take place. It is known that insulin acts on the whole body reducing glucose and triglyceride levels [49], and increasing fat mass. Since BDNF can increase peripheral insulin sensitivity [50], we hypothesize that, in CCT group, the lack of increase in BDNF could lead to a global anti-insular effect on the whole body, resulting in an additional beneficial effect for the brain in the short term. Indeed, the increase of plasma glucose guarantees high levels of substrate for brain metabolism; furthermore, since there is not increase in insulin sensitivity, the nutrients are preserved from the consumption by the muscle or from the storage in adipose tissue. In other terms, in CCT group, where at the end of bed rest BDNF levels are not increased, the negative effects of bed rest on peripheral metabolism can be a temporary effort to protect the brain from insults. On the opposite, in noCCT group, the increase of BDNF not only acts in the brain, but its metabolic effects take place in the whole body, ameliorating the insulin sensitivity. One could wonder which one of these conditions is better for the individual: preserving the brain despite a metabolic stress for the whole body or ameliorating the metabolic profile. To answer, we evaluated muscle tropism/neuromuscular function in both groups.

### Bed rest, computerized cognitive training, and neuromuscular parameters

We have previously shown that during two weeks of bed rest, CCT was effective and durable: subjects who underwent CCT performed significantly better on maze navigation at least 1.5 years after training [29]. Participants who underwent CCT also showed transfer of training: enhanced executive functioning, attention and processing speed. Moreover, even though there was a detectable decline in the ability to perform a complex motor-cognitive dual-tasks in the control group of older adults, CCT reduced the negative impact of bed rest on these integrated tasks [31]. More specifically, CCT prevented the decline in fast pace dual-task walking condition and positively affected gait variability [31]. These results indicate CCT as a strong protective mechanism regarding integrated tasks requiring contemporaneous activation of motor and cognitive functions.

We evaluated whether the muscle mechanical output, expected to worsen at the end of bed rest, could benefit from CCT too. As in previous studies [46-48, 51], bed rest induced a decrease in muscle mass and lower limb muscle function. Quantitative muscular parameters were similar in the two groups; only FFM, an indirect measure of muscle mass, was significantly different. However, considering the difference in homogeneity of the sample, we must acknowledge that this result could have statistical, but not clinical relevance. Similarly, analyzing functional parameters, the difference in MVC of the knee extensors seems to derive from the different homogeneity of results within the groups, and not from a real clinical difference. However, interesting results come from correlation analysis between BDNF plasma levels and MEP, in which CCT and noCCT group show opposite behaviors. MEP depends firstly on muscular mass and neural drive/motor unit recruitment pattern, processes known to be regulated by BDNF. Namely, BDNF is involved in the processes of neural plasticity in the hippocampus and in spinal cord [52], and, in spinally transected rats, the protective and restorative effect of “instrumental brain training” was demonstrated to be attributable to the increase of lumbar spinal cord BDNF [53]. The finding of a positive correlation between plasma BDNF and MEP in noCCT group is in line with these results, and might indicate that BDNF increase protects motor unit from detrimental effects of bed rest. The protective role of BDNF in noCCT group is also confirmed by the observation that ΔBDNF negatively correlates with ΔMVC in this group, which means that the more BDNF levels increase, the less MVC is lost, pointing to the preservation of muscular function.

On the opposite, CCT group show a negative correlation between plasma BDNF and MEP. This apparently paradoxical situation could be explained by the “brain efficiency hypothesis”: it was already demonstrated that young healthy people undergoing brain training show structural changes in the frontal lobe, which were inversely correlated with the degree of improvement of performance across training session (the greater the changes the lower the improvement in cognitive performance) [54]. This was interpreted considering that higher performers have the required resources for successfully coping with the cognitive challenges comprised by training, whereas that was not the case in low performers; the lack of processing resources might evoke the greater changes observed in the brain structure. Similarly, we can hypothesize that, in people lacking the necessary resources, an increase in BDNF might take place to cope with the challenge. People with higher BDNF should be less “performing”, and this could explain why in this group the higher BDNF correlates with the lower MEP.

In conclusion, in our hypothesis, BDNF increase is an ancestral, standardized protective mechanism that takes place whenever an acute insult occurs. CCT, acting as an external protective mechanism, modifies this standardized response, reducing the increase of BDNF or possibly only modifying the time course of BDNF response. Elucidating the clear pathway of the differential response to bed rest stress in presence/absence of CCT goes beyond the objectives of this study, therefore future studies are needed to verify our hypothesis. Nevertheless, the present findings emphasize the need to study the different mechanisms involved in neuroprotection acting in healthy and ill individuals, underlines the possible importance of timing in determining the effects of protective mechanisms, and points out some concepts:

### Limitations of this study

We must finally acknowledge some limitations of this study. The first limit is the dimension of the sample. Despite being a very standardized and controlled (“in vivo study”), our study was performed on a small number of individuals, and this makes it difficult to drive any unequivocal conclusion. However, it is necessary to underline that bed rest studies including older individuals are rare, and that bed rest studies are very costly, labor intensive, and limited by hospital capacity. The total costs are estimated over $20,000 per participant. However, despite having such a small population, we could detect variables which were correlated with moderate significance. We hypothesize that in a larger population even stronger statistical significance could be reached. For the same financial and logistic reasons, it was not possible to obtain blood samples during the different phases of bed rest and CCT, to further sustain our hypothesis. However, it is our intention to perform in future another study to evaluate this aspect.

Third, since we did not perform functional neuroimaging and did not obtain cerebro-spinal fluid from subjects, we can evaluate “brain stress” only by means of surrogate markers, such as the worsening of integrated functions; similarly, not showing results from muscular biopsy, we can only discuss surrogate variables for muscular quality.

## METHODS

### Population

We designed a study composed by two groups of healthy volunteers: the Computerized Cognitive Training (CCT) group (8 subjects, mean age 59.1 ± 3.6 years) and a no-Computerized Cognitive Training (noCCT) (8 subjects, mean age 59.1 ± 2.5 years). After 3 days of ambulatory period (regulated hospital diet and daily activities) all subjects underwent horizontal bed rest for 14 days in standard air-conditioned hospital rooms of the Orthopedic Hospital of Valdoltra (Slovenia). During the whole bed rest procedure, constant surveillance and 24-hour medical care was provided and all subjects received an individually controlled normo-caloric diet: for each subject resting energy expenditure (estimated by means of bio-impedentiometric- BIA measures) was multiplied by factor 1.2, with 60% caloric content coming from carbohydrates, 25% from fat and 15% from proteins. Subjects performed all daily activities in bed, were allowed to freely communicate, watch television and listen to radio, read, use computer and to receive visitors. The members of CCT group also performed daily CCT intervention by navigating through virtual mazes using a joystick device (for detailed protocol see [31]); the members of the noCCT group watched documentaries at the same time and for the same amount of time while CCT performed brain training.

Exclusion criteria were: smoking; regular alcohol consumption; ferromagnetic implants; history of deep vein thrombosis with D-dimer levels at enrolment greater than 500 μg·L-1; acute or chronic skeletal, neuromuscular, metabolic and cardiovascular disease conditions; pulmonary embolism. All subjects gave their written informed consent. The study was performed in accordance with the ethical standards of the 1964 Declaration of Helsinki.

### Computerized cognitive training

All members of CCT group performed 50 minutes computerized cognitive training daily from the second to the thirteenth day of bed rest. As reported in our previous work [31], the CCT consisted of moving through virtual environments using a Trust Predator Joystick GM- 2550. Mazes were presented on a 17-in. flat-panel LCD monitor situated approximately 60 cm in front of each participant. Mazes represented a series of interconnected corridors, with three available paths at each intersection or decision-making point, with either a pair of verbal (signs with country names, city names, and animal names) or pictorial (country flags, animal pictures, and human faces) cues displayed at either opposite corner of the intersection and in corridors. Participants were instructed to select the correct path as quickly and efficiently as possible in order to virtually walk toward the goal area. Mazes were of increasing difficulty, designed using a modified version of Unreal Tournament 2003 and the Unreal Editor 3.0 (Epic Games, Inc.) software package [55, 56].

For more detailed explanation of CCT procedure, readers are referred to [29].

### Biologic samples

Blood samples were collected from each subject at enrolment (baseline data collection - BDC) and after 14 days of forced bed rest (BR14), after an overnight fasting and centrifuged immediately after blood collection. Aliquots were stored at -80°C.

HDL cholesterol after precipitation of the apo-B containing lipoproteins [57], total cholesterol and triglycerides (TG) levels were assayed in serum by the Trinder method. The coefficient of variation was < 2% for Total and HDL cholesterol and < 5% for TG for intra- and inter-batch, respectively. LDL-C plasma levels were calculated by the Friedewald's formula [58]. Plasma glucose was measured using standard enzymatic methods (FAR S.R.L., Italy). The coefficient of variation was < 3% for intra-assay. Fasting insulin levels were assayed using an ultrasensitive insulin ELISA kit manufactured by Mercodia AB (Sweden). The coefficient of variation was < 3% for intra-assay. Fasting insulin resistance was evaluated calculating Homeostasis Model Assessment (HOMA) [59]. TNF-alpha plasma levels was measured using an ELISA Kit (Invitrogen). The coefficient of variation was < 5.2% for intra-assay. C-reactive protein plasma levels were measured using an immunoturbidimetric test (High sensitive C-Reactive protein, Roche. Italy). The coefficient of variation was < 2% for intra-assay. BDNF plasma levels were measured by means of ELISA (Promega Italia S.r.l, Italy), following the manufacturer's instructions.

### Anthropometric characteristics and body composition

Body mass (BM) was measured to the nearest 0.1 kg with a manual weighing scale (Seca 709, Hamburg, Germany) with the subject dressed only in light underwear and no shoes. Stature was measured to the nearest 0.5 cm on a standardized wall-mounted height board.

Body composition was measured by using bioelectrical impedance analysis with a tetra-polar impedance-meter (BIA101, Akern, Florence, Italy), according to the method of Lukasky et al. [60]. Body composition (fat-free mass, FFM, fat mass, FM, Muscle Mass, MM, and Body Cellular Mass, BCM) was obtained from the software provided by the manufacturer. This method has been already utilized and validated to investigate changes in body composition during BR and in clinical practice [61]. For each participant these measures were obtained both at BDC and BR14, after eight hours fasting.

### Magnetic resonance imaging

Quadriceps muscle volume of the right limb was measured from turbo spin-echo, T1-weighted, magnetic resonance images (MRI) obtained with 1.5 T (Magnetom Avanto; Siemens Medical Solution, Erlangen, Germany). As reported in our previous work [62], on each MRI slice, contours corresponding to the quadriceps muscles were delineated by an expert of MRI imaging, using the image processing tools OsiriX (Pixmeo Sarl, v.4.1.2). Quadriceps muscle volume was then derived by summation of a series of evenly spaced truncated cones comprised between each two axial images, a process that included an average of 25 images (range 23-28) and covered the entire length of the quadriceps.

### Maximal explosive power of the lower limbs

The biomechanical parameters of the explosive efforts were studied by means of the Explosive Ergometer (EXER), described previously in details [62, 63]. Briefly, EXER consists of a metal frame supporting one rail, which was inclined by 20 degrees. A seat, fixed on a carriage, was free to move on the rail, its velocity along the direction of motion being continuously recorded by a wire tachometer (LIKA SGI, Vicenza, Italy). The subject was able to accelerate himself and the carriage seat backward pushing on two force platforms (LAUMAS PA 300, Parma, Italy) positioned perpendicular to the rail. The total moving mass of the EXER (seat and carriage together) was equal to 31.6 kg. Force and velocity analog outputs were sampled at 2000 Hz using a data acquisition system (MP100; BIOPAC Systems, Inc., Goleta, CA, USA). The instantaneous power was calculated from the product of instantaneous force and velocity values.

The subject was seated on the carriage seat, secured by a safety harness tightened around the shoulders and abdomen, with his arms on handlebars. Two mechanical blocks were used to set the distance between the seat and the force platforms, so that the knee angle at rest was 110 degrees. The blocks also prevented any counter-movement and, consequently, any recovery of elastic energy during the pushing phase. After a brief familiarisation session with the laboratory equipment, the subjects performed four maximal explosive efforts, the duration of which was about 400 ms. After each push, subjects rested for 2 minutes with their feet placed on a support. The attempt with the greatest peak power (maximal explosive power, MEP) was taken into account for further analysis.

### Maximal voluntary contractions

Maximal voluntary isometric contractions (MVCs) of the right knee extensors were performed on a special chair where the subject was seated with the legs hanging vertically down [64]. A strap, connected in series to a force sensor (TSD121C, BIOPAC Systems, Inc., Goleta, CA), was tightened around the subject's right ankle. The force sensor was fixed in series to a steel frame, the position of which was set in order to obtain a knee angle of 110 degrees. Force exerted during MVC was recorded at a frequency of 2 kHz using a data acquisition system (MP100, BIOPAC Systems, Inc., Goleta, CA). Subjects were asked to perform three MVCs of four – five seconds each. To prevent fatigue, after each contraction subjects rested for two minutes.

### Statistical analysis

Continuous variables were expressed as mean (Standard Deviation) or, when necessary, as median (interquartile range). Categorical variables were expressed as number/percentage. At BDC, means were compared by one way ANOVA, while medians were compared by non-parametric tests (Kruskal Wallis). Normality of distribution was tested with Shapiro-Wilk test. Variations between BDC and BR14 in BDNF plasma levels and the other variables of interest were analysed by T-test for repeated measures and General Linear Model (GLM) Repeated Measures, Within-Subjects and Between-Subjects test.

Correlations between continuous variables were tested by Pearson's correlation test for variables with normal distribution. Variables with non-normal distribution were analyzed after log transformation or with non-parametric test (Spearman's test).

Statistical analysis was performed using SPSS 22.0 software (SPSS Inc., Chicago, IL, USA) and statistical significance was set a P < 0.05.
